# 1,4-Bis{(+)-(*S*)-[1-(1-naphth­yl)eth­yl]imino­meth­yl}benzene

**DOI:** 10.1107/S1600536809033455

**Published:** 2009-09-05

**Authors:** Armando Espinosa Leija, Sylvain Bernès, Guadalupe Hernández, Pankaj Sharma, Ulises Peña, René Gutiérrez

**Affiliations:** a Facultad de Ciencias Químicas, UANL, Licenciatura en Química Industrial, Ciudad Universitaria, Monterrey, NL, Mexico; b DEP Facultad de Ciencias Químicas, UANL, Guerrero y Progreso S/N, Col. Treviño, 64570 Monterrey, NL, Mexico; c Lab. Síntesis de Complejos, Facultad de Ciencias Químicas, Universidad Autónoma de Puebla, PO Box 1067, 72001 Puebla, Pue., Mexico; d Instituto de Química–UNAM, Circuito exterior, Cd. Universitaria, Coyoacán, CP 04510, México, DF, Mexico

## Abstract

The title compound, C_32_H_28_N_2_, is a chiral bis-imine in which both imine groups display the common *E* configuration. The naphthyl groups present different orientations with respect to the central core, as reflected in the dihedral angles of 21.4 (2) and 78.83 (14)° between the benzene and naphthyl mean planes, thus the highest possible *C*_2_ local molecular symmetry is not attained. This *C*_1_ mol­ecular conformation allows multiple C—H⋯π inter­molecular contacts involving all aromatic rings, while no π–π inter­actions are available for the stabilization of the crystal structure. The resulting packing structure is based on mol­ecules stacked along [100].

## Related literature

For solvent-free synthesis in organic chemistry, see: Jeon *et al.* (2005 ▶); Noyori (2005 ▶); Tanaka & Toda (2000 ▶); Tovar *et al.* (2007 ▶). For related chiral Schiff bases constructed from a bis-substituted benzene core, see: Allouchi *et al.* (1994 ▶); Hamaker & Oberts (2006 ▶); Espinosa Leija *et al.* (2009 ▶). For the use of the enanti­omer of the title compound as a chiral dopant for liquid crystals, see: Watanabe & Fukuda (2008 ▶).
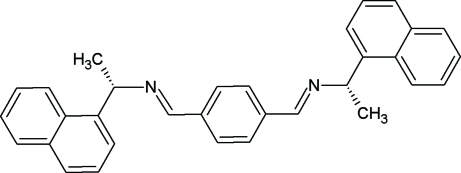


## Experimental

### 

#### Crystal data

C_32_H_28_N_2_
*M* *_r_* = 440.56
Orthorhombic, 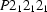

*a* = 8.391 (3) Å
*b* = 15.102 (5) Å
*c* = 19.569 (7) Å
*V* = 2479.6 (14) Å^3^
*Z* = 4
Mo *K*α radiation
μ = 0.07 mm^−1^
*T* = 298 K
0.6 × 0.2 × 0.2 mm

#### Data collection

Siemens P4 diffractometer
Absorption correction: none
6140 measured reflections
2491 independent reflections
1445 reflections with *I* > 2σ(*I*)
*R*_int_ = 0.162
3 standard reflections every 97 reflections intensity decay: 2.5%

#### Refinement

*R*[*F* ^2^ > 2σ(*F* ^2^)] = 0.052
*wR*(*F* ^2^) = 0.163
*S* = 1.10
2491 reflections
310 parameters
H-atom parameters constrained
Δρ_max_ = 0.18 e Å^−3^
Δρ_min_ = −0.18 e Å^−3^

### 

Data collection: *XSCANS* (Siemens, 1996 ▶); cell refinement: *XSCANS*; data reduction: *XSCANS*; program(s) used to solve structure: *SHELXS97* (Sheldrick, 2008 ▶); program(s) used to refine structure: *SHELXL97* (Sheldrick, 2008 ▶); molecular graphics: *Mercury* (Macrae *et al.*, 2006 ▶); software used to prepare material for publication: *SHELXL97*.

## Supplementary Material

Crystal structure: contains datablocks I, global. DOI: 10.1107/S1600536809033455/lh2874sup1.cif

Structure factors: contains datablocks I. DOI: 10.1107/S1600536809033455/lh2874Isup2.hkl

Additional supplementary materials:  crystallographic information; 3D view; checkCIF report

## Figures and Tables

**Table 1:** Hydrogen-bond geometry (Å, °)

*D*—H⋯*A*	*D*—H	H⋯*A*	*D*⋯*A*	*D*—H⋯*A*
C12—H12*A*⋯*Cg*3^i^	0.96	2.79	3.677 (6)	154
C18—H18*A*⋯*Cg*4^i^	0.93	2.62	3.520 (5)	163
C20—H20*A*⋯*Cg*5^i^	0.93	2.98	3.681 (5)	133

Symmetry code: (i) 
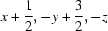
. *Cg*1 is the centroid of ring C27–C32, *Cg*2 is the centroid of ring C23–C27/C32, *Cg*3 is the centroid of ring C14–C19, *Cg*4 is the centroid of ring C1–C5/C10 and *Cg*5 is the centroid of ring C5–C10.

**Fig. 1.**
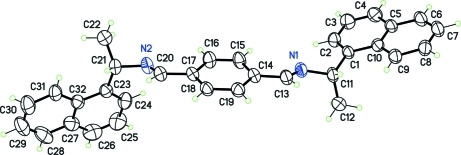
The title molecule with displacement ellipsoids for non-H atoms shown at the 30% probability level.

**Fig. 2.**
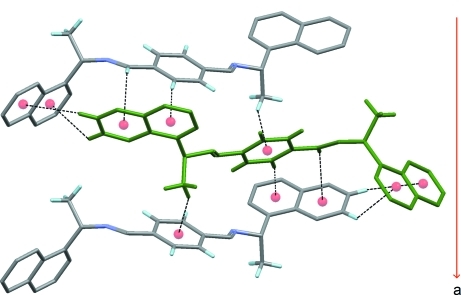
A part of the crystal structure of the title compound, with the asymmetric unit shown in green. Dashed lines represent C—H···π interactions in the crystal, and centroids of involved π systems have been represented with red spheres. Some H atoms not involved in the network of contacts have been omitted for clarity.

## supplementary crystallographic information

### Comment

There is an increased interest in the use of environmentally benign reagents and conditions particularly to solvent-free procedures. Thus, avoiding organic solvents during the reactions in organic synthesis leads to clean, efficient and economical technology: safety is largely increased, working is considerably simplified, cost is reduced, increased amounts of reactants can be used, *etc*. Also, reactivities and sometimes selectivities are enhanced (Jeon *et al.*, 2005; Noyori, 2005; Tanaka & Toda, 2000). On the other hand, bis-imines have lately attracted much attention, mostly due to their versatile coordination behavior and the interesting properties of their metal complexes. These compounds are particularly interesting since they can potentially act in a variety of coordination modes. Continuing our work on the synthesis and characterization of this kind of compounds (Tovar *et al.*, 2007; Espinosa Leija *et al.*, 2009), we synthesized the title compound under solvent-free conditions and report herein its crystal structure.

The molecule (Fig. 1) is constructed of a benzene ring *para*-substituted by two identical chiral fragments including imine functionality. The conformation stabilized in the solid-state has both imine groups displaying *E* configuration, previously observed in related systems (*e.g*. Allouchi *et al.*, 1994). Naphthyl groups, which are potentially free to rotate about their σ bonds C1—C11 and C21—C23, show different orientations with respect to the central benzene ring. The dihedral angles between the central benzene ring C14···C19 and the naphthyl rings C1···C10 and C23···C32 are 21.4 (2) and 78.83 (14)°, respectively. The naphthyl systems make a dihedral angle of 73.69 (10)°. As a consequence, the molecule has *C*_1_ point symmetry rather than *C*_2_, and is not a good candidate for coordination to transition metals. In contrast, other related bis-imines based on a *para*-substituted benzene core approximate the *C*_2_ point symmetry (*e.g*. Hamaker & Oberts, 2006).

The crystal structure features a number of C—H···π intermolecular interactions of variable strength, involving all available aromatic rings (Fig. 2). Although no π–π contacts contribute to the stabilization of the crystal structure, the molecules are efficiently packed along the short [100] axis in the crystal. As a consequence, no voids are available for lattice solvent insertion, a situation contrasting with that observed for an isomeric system previously described (Espinosa Leija *et al.*, 2009): for the *meta*-substituted molecule, a 1:1 solvate was crystallized with CH_2_Cl_2_, with solvent molecules filling large voids generated by the molecular conformation.

Interestingly, the enantiomer of the title compound has been registered (Watanabe & Fukuda, 2008; CAS registry number: 1021327–88-7) as a chiral dopant for nematic or cholesteric liquid crystals for generating large helical twisting power. This use is consistent with the high optical rotation measured for this molecule (see *Experimental*).

### Experimental

Under solvent-free conditions, a mixture of benzene-1,4-dicarboxaldehyde (0.12 g, 0.93 mmol) and (*S*)-(-)-1-naphthylethylamine (0.32 g, 1.8 mmol) were mixed at 298 K, giving a white solid. The crude material was recrystallized twice from CH_2_Cl_2_, affording colorless crystals suitable for X-ray diffraction. Yield: 87%; m.p. 438 K (165 °C); [α]_D_^25^ = +413.3 (*c* 1, CHCl_3_). IR (KBr): 1632 cm^-1^ (C═N). ^1^H-NMR (400 MHz, CDCl_3_/TMS): δ = 1.73 (d, 6 H, CHC*H*_3_), 5.35 (q, 2H, C*H*), 7.45–8.24 (m, 18 H, Ar), 8.42 (s, 2 H, *H*C=N). ^13^C-NMR (100 MHz, CDCl_3_/TMS) δ = 24.4 (C*C*H_3_), 65.6 (*C*HCH_3_), 123.5 (Ar), 124.0 (Ar), 125.3 (Ar), 125.6 (Ar), 125.8 (Ar), 127.3 (Ar), 128.4 (Ar), 128.9 (Ar), 130.5 (Ar), 133.9 (Ar), 138.3 (Ar), 140.9 (Ar), 159.1 (H*C*=N). MS—EI: *m/z*= 440 (*M*^+^) for C_32_H_28_N_2_.

### Refinement

All H atoms were placed in idealized positions with C—H bond lengths fixed to 0.93 (aromatic), 0.96 (methyl) or 0.98 Å (methine), and with methyl groups allowed to rotate about their C—C bonds. A riding refinement was applied, and isotropic displacement parameters were computed as *U*_iso_(H) = 1.5*U*_eq_(carrier atom) for the methyl groups and *U*_iso_(H) = 1.2*U*_eq_(carrier atom) otherwise. Friedel pairs (1571) were merged and the absolute configuration inferred from that of the commercial optically pure amine used as starting material.

### Crystal data

**Table:**

C_32_H_28_N_2_	*D*_x_ = 1.180 Mg m^−^^3^
*M**_r_* = 440.56	Melting point: 438 K
Orthorhombic, *P*2_1_2_1_2_1_	Mo *K*α radiation, λ = 0.71073 Å
Hall symbol: P 2ac 2ab	Cell parameters from 100 reflections
*a* = 8.391 (3) Å	θ = 4.8–11.4°
*b* = 15.102 (5) Å	µ = 0.07 mm^−^^1^
*c* = 19.569 (7) Å	*T* = 298 K
*V* = 2479.6 (14) Å^3^	Needle, colorless
*Z* = 4	0.6 × 0.2 × 0.2 mm
*F*(000) = 936	

### Data collection

**Table:**

Siemens P4 diffractometer	*R*_int_ = 0.162
Radiation source: fine-focus sealed tube	θ_max_ = 25.1°, θ_min_ = 2.1°
graphite	*h* = −9→6
ω scans	*k* = −17→17
6140 measured reflections	*l* = −23→22
2491 independent reflections	3 standard reflections every 97 reflections
1445 reflections with *I* > 2σ(*I*)	intensity decay: 2.5%

### Refinement

**Table:**

Refinement on *F*^2^	Secondary atom site location: difference Fourier map
Least-squares matrix: full	Hydrogen site location: inferred from neighbouring sites
*R*[*F*^2^ > 2σ(*F*^2^)] = 0.052	H-atom parameters constrained
*wR*(*F*^2^) = 0.163	*w* = 1/[σ^2^(*F*_o_^2^) + (0.0396*P*)^2^ + 0.384*P*] where *P* = (*F*_o_^2^ + 2*F*_c_^2^)/3
*S* = 1.10	(Δ/σ)_max_ < 0.001
2491 reflections	Δρ_max_ = 0.18 e Å^−^^3^
310 parameters	Δρ_min_ = −0.18 e Å^−^^3^
0 restraints	Extinction correction: *SHELXL97* (Sheldrick, 2008), Fc^*^=kFc[1+0.001xFc^2^λ^3^/sin(2θ)]^-1/4^
0 constraints	Extinction coefficient: 0.020 (3)
Primary atom site location: structure-invariant direct methods	

### Fractional atomic coordinates and isotropic or equivalent isotropic displacement parameters (Å^2^)

**Table:**

	*x*	*y*	*z*	*U*_iso_*/*U*_eq_	
N1	0.1296 (6)	0.6293 (2)	0.04836 (18)	0.0727 (12)	
N2	0.0862 (6)	1.0684 (2)	0.17915 (19)	0.0772 (13)	
C1	0.0418 (6)	0.4748 (2)	0.0380 (2)	0.0627 (12)	
C2	−0.0244 (7)	0.4806 (3)	0.1019 (2)	0.0737 (14)	
H2A	−0.0007	0.5292	0.1292	0.088*	
C3	−0.1255 (8)	0.4159 (3)	0.1265 (3)	0.0870 (17)	
H3A	−0.1694	0.4219	0.1699	0.104*	
C4	−0.1613 (8)	0.3441 (3)	0.0885 (3)	0.0872 (17)	
H4A	−0.2284	0.3007	0.1061	0.105*	
C5	−0.0977 (7)	0.3344 (3)	0.0223 (3)	0.0708 (13)	
C6	−0.1344 (8)	0.2612 (3)	−0.0191 (3)	0.0862 (17)	
H6A	−0.1986	0.2165	−0.0014	0.103*	
C7	−0.0796 (8)	0.2534 (3)	−0.0837 (3)	0.0876 (17)	
H7A	−0.1063	0.2042	−0.1099	0.105*	
C8	0.0173 (8)	0.3197 (3)	−0.1110 (3)	0.0799 (16)	
H8A	0.0528	0.3154	−0.1559	0.096*	
C9	0.0604 (7)	0.3912 (3)	−0.0719 (2)	0.0708 (13)	
H9A	0.1276	0.4340	−0.0903	0.085*	
C10	0.0046 (6)	0.4010 (2)	−0.0043 (2)	0.0633 (12)	
C11	0.1588 (7)	0.5439 (3)	0.0144 (3)	0.0733 (14)	
H11A	0.1496	0.5514	−0.0352	0.088*	
C12	0.3255 (7)	0.5134 (3)	0.0319 (4)	0.107 (2)	
H12A	0.4009	0.5576	0.0179	0.161*	
H12B	0.3477	0.4589	0.0085	0.161*	
H12C	0.3337	0.5043	0.0803	0.161*	
C13	0.1601 (7)	0.6977 (3)	0.0141 (2)	0.0708 (14)	
H13A	0.1931	0.6908	−0.0310	0.085*	
C14	0.1463 (7)	0.7871 (3)	0.0412 (2)	0.0643 (13)	
C15	0.0690 (8)	0.8039 (3)	0.1020 (2)	0.0793 (16)	
H15A	0.0209	0.7576	0.1257	0.095*	
C16	0.0622 (8)	0.8888 (3)	0.1283 (2)	0.0784 (16)	
H16A	0.0113	0.8990	0.1697	0.094*	
C17	0.1308 (7)	0.9588 (3)	0.0932 (2)	0.0642 (12)	
C18	0.2021 (7)	0.9427 (3)	0.0316 (2)	0.0670 (13)	
H18A	0.2450	0.9894	0.0067	0.080*	
C19	0.2112 (7)	0.8571 (3)	0.0060 (2)	0.0688 (13)	
H19A	0.2618	0.8471	−0.0356	0.083*	
C20	0.1253 (7)	1.0499 (3)	0.1189 (2)	0.0695 (14)	
H20A	0.1517	1.0959	0.0894	0.083*	
C21	0.0880 (7)	1.1625 (3)	0.1993 (2)	0.0719 (14)	
H21A	0.1013	1.1994	0.1585	0.086*	
C22	−0.0707 (8)	1.1827 (3)	0.2317 (3)	0.0884 (16)	
H22A	−0.1543	1.1716	0.1994	0.133*	
H22B	−0.0735	1.2437	0.2454	0.133*	
H22C	−0.0853	1.1455	0.2711	0.133*	
C23	0.2264 (7)	1.1784 (3)	0.2475 (2)	0.0694 (14)	
C24	0.2964 (9)	1.1093 (3)	0.2803 (2)	0.0887 (17)	
H24A	0.2575	1.0524	0.2729	0.106*	
C25	0.4234 (11)	1.1211 (5)	0.3240 (3)	0.116 (2)	
H25A	0.4690	1.0724	0.3455	0.139*	
C26	0.4811 (10)	1.2020 (5)	0.3357 (3)	0.115 (2)	
H26A	0.5665	1.2092	0.3655	0.138*	
C27	0.4139 (9)	1.2771 (4)	0.3034 (3)	0.0903 (18)	
C28	0.4724 (11)	1.3636 (5)	0.3141 (3)	0.117 (3)	
H28A	0.5584	1.3721	0.3433	0.141*	
C29	0.4064 (11)	1.4349 (4)	0.2826 (4)	0.115 (3)	
H29A	0.4469	1.4913	0.2903	0.137*	
C30	0.2805 (10)	1.4230 (4)	0.2398 (3)	0.102 (2)	
H30A	0.2352	1.4718	0.2184	0.123*	
C31	0.2197 (8)	1.3413 (3)	0.2279 (2)	0.0793 (15)	
H31A	0.1339	1.3351	0.1981	0.095*	
C32	0.2831 (7)	1.2664 (3)	0.2593 (2)	0.0710 (14)	

### Atomic displacement parameters (Å^2^)

**Table:**

	*U*^11^	*U*^22^	*U*^33^	*U*^12^	*U*^13^	*U*^23^
N1	0.082 (3)	0.0544 (19)	0.081 (2)	−0.010 (2)	0.003 (2)	−0.0083 (18)
N2	0.104 (4)	0.056 (2)	0.072 (2)	−0.013 (2)	0.002 (3)	−0.0035 (18)
C1	0.066 (3)	0.047 (2)	0.076 (3)	−0.007 (2)	−0.006 (3)	0.000 (2)
C2	0.086 (4)	0.061 (2)	0.074 (3)	−0.004 (3)	0.004 (3)	−0.001 (2)
C3	0.098 (5)	0.081 (3)	0.082 (3)	−0.013 (4)	0.011 (3)	0.009 (3)
C4	0.088 (4)	0.072 (3)	0.102 (4)	−0.015 (3)	0.012 (4)	0.012 (3)
C5	0.064 (3)	0.051 (2)	0.098 (3)	−0.003 (2)	−0.012 (3)	−0.001 (2)
C6	0.082 (4)	0.055 (3)	0.122 (5)	−0.007 (3)	−0.020 (4)	−0.008 (3)
C7	0.089 (4)	0.061 (3)	0.112 (4)	−0.001 (3)	−0.027 (4)	−0.019 (3)
C8	0.085 (4)	0.068 (3)	0.087 (3)	0.015 (3)	−0.020 (3)	−0.019 (3)
C9	0.074 (3)	0.060 (2)	0.078 (3)	0.001 (3)	−0.009 (3)	−0.004 (2)
C10	0.063 (3)	0.051 (2)	0.076 (3)	0.005 (2)	−0.013 (3)	−0.001 (2)
C11	0.086 (4)	0.052 (2)	0.082 (3)	−0.012 (3)	0.009 (3)	−0.013 (2)
C12	0.075 (4)	0.081 (3)	0.166 (6)	−0.016 (3)	0.017 (4)	−0.026 (4)
C13	0.081 (4)	0.058 (2)	0.074 (3)	−0.010 (3)	0.001 (3)	−0.008 (2)
C14	0.074 (4)	0.054 (2)	0.065 (2)	−0.012 (3)	−0.004 (3)	−0.003 (2)
C15	0.109 (5)	0.060 (3)	0.069 (3)	−0.020 (3)	0.012 (3)	0.001 (2)
C16	0.106 (5)	0.065 (3)	0.064 (3)	−0.012 (3)	0.013 (3)	−0.004 (2)
C17	0.070 (3)	0.053 (2)	0.070 (2)	−0.012 (2)	−0.003 (3)	−0.003 (2)
C18	0.071 (3)	0.055 (2)	0.074 (3)	−0.015 (3)	0.011 (3)	0.000 (2)
C19	0.073 (3)	0.060 (2)	0.073 (3)	−0.008 (3)	0.008 (3)	0.001 (2)
C20	0.078 (4)	0.056 (2)	0.074 (3)	−0.009 (3)	−0.001 (3)	0.001 (2)
C21	0.089 (4)	0.055 (2)	0.072 (3)	−0.003 (3)	0.001 (3)	−0.006 (2)
C22	0.085 (4)	0.082 (3)	0.099 (4)	−0.002 (3)	−0.002 (3)	−0.002 (3)
C23	0.075 (4)	0.070 (3)	0.063 (3)	−0.004 (3)	0.003 (3)	−0.004 (2)
C24	0.113 (5)	0.076 (3)	0.077 (3)	0.009 (4)	−0.004 (4)	0.005 (3)
C25	0.126 (7)	0.123 (5)	0.098 (4)	0.022 (5)	−0.037 (5)	0.011 (4)
C26	0.104 (6)	0.159 (6)	0.082 (4)	0.009 (6)	−0.027 (4)	−0.007 (4)
C27	0.087 (5)	0.109 (4)	0.075 (3)	−0.011 (4)	−0.002 (3)	−0.018 (3)
C28	0.120 (6)	0.144 (6)	0.089 (4)	−0.056 (6)	−0.008 (4)	−0.034 (4)
C29	0.147 (8)	0.094 (4)	0.103 (4)	−0.046 (5)	0.024 (5)	−0.029 (4)
C30	0.139 (7)	0.080 (3)	0.088 (3)	−0.017 (4)	0.020 (4)	−0.018 (3)
C31	0.098 (4)	0.065 (3)	0.076 (3)	−0.005 (3)	0.006 (3)	−0.013 (2)
C32	0.076 (4)	0.073 (3)	0.064 (3)	−0.005 (3)	0.003 (3)	−0.014 (2)

### Geometric parameters (Å, °)

**Table:**

N1—C13	1.257 (5)	C15—H15A	0.9300
N1—C11	1.472 (5)	C16—C17	1.386 (6)
N2—C20	1.255 (5)	C16—H16A	0.9300
N2—C21	1.476 (5)	C17—C18	1.367 (6)
C1—C2	1.371 (6)	C17—C20	1.465 (5)
C1—C10	1.423 (5)	C18—C19	1.388 (5)
C1—C11	1.505 (6)	C18—H18A	0.9300
C2—C3	1.380 (7)	C19—H19A	0.9300
C2—H2A	0.9300	C20—H20A	0.9300
C3—C4	1.350 (7)	C21—C22	1.506 (8)
C3—H3A	0.9300	C21—C23	1.515 (7)
C4—C5	1.409 (7)	C21—H21A	0.9800
C4—H4A	0.9300	C22—H22A	0.9600
C5—C6	1.406 (6)	C22—H22B	0.9600
C5—C10	1.420 (6)	C22—H22C	0.9600
C6—C7	1.350 (8)	C23—C24	1.359 (7)
C6—H6A	0.9300	C23—C32	1.430 (6)
C7—C8	1.396 (7)	C24—C25	1.378 (10)
C7—H7A	0.9300	C24—H24A	0.9300
C8—C9	1.371 (6)	C25—C26	1.335 (8)
C8—H8A	0.9300	C25—H25A	0.9300
C9—C10	1.412 (6)	C26—C27	1.415 (8)
C9—H9A	0.9300	C26—H26A	0.9300
C11—C12	1.511 (8)	C27—C32	1.406 (8)
C11—H11A	0.9800	C27—C28	1.412 (8)
C12—H12A	0.9600	C28—C29	1.358 (9)
C12—H12B	0.9600	C28—H28A	0.9300
C12—H12C	0.9600	C29—C30	1.360 (11)
C13—C14	1.456 (5)	C29—H29A	0.9300
C13—H13A	0.9300	C30—C31	1.355 (7)
C14—C19	1.375 (6)	C30—H30A	0.9300
C14—C15	1.379 (6)	C31—C32	1.393 (7)
C15—C16	1.383 (6)	C31—H31A	0.9300
			
C13—N1—C11	116.4 (4)	C17—C16—H16A	119.8
C20—N2—C21	117.5 (4)	C18—C17—C16	118.8 (4)
C2—C1—C10	119.4 (4)	C18—C17—C20	118.9 (4)
C2—C1—C11	120.0 (4)	C16—C17—C20	122.2 (4)
C10—C1—C11	120.5 (4)	C17—C18—C19	120.6 (4)
C1—C2—C3	121.5 (4)	C17—C18—H18A	119.7
C1—C2—H2A	119.2	C19—C18—H18A	119.7
C3—C2—H2A	119.2	C14—C19—C18	120.9 (4)
C4—C3—C2	120.9 (5)	C14—C19—H19A	119.6
C4—C3—H3A	119.6	C18—C19—H19A	119.6
C2—C3—H3A	119.6	N2—C20—C17	122.6 (4)
C3—C4—C5	120.4 (5)	N2—C20—H20A	118.7
C3—C4—H4A	119.8	C17—C20—H20A	118.7
C5—C4—H4A	119.8	N2—C21—C22	107.3 (5)
C6—C5—C4	121.9 (5)	N2—C21—C23	109.1 (4)
C6—C5—C10	118.6 (5)	C22—C21—C23	112.5 (4)
C4—C5—C10	119.5 (4)	N2—C21—H21A	109.3
C7—C6—C5	122.3 (5)	C22—C21—H21A	109.3
C7—C6—H6A	118.9	C23—C21—H21A	109.3
C5—C6—H6A	118.9	C21—C22—H22A	109.5
C6—C7—C8	119.6 (5)	C21—C22—H22B	109.5
C6—C7—H7A	120.2	H22A—C22—H22B	109.5
C8—C7—H7A	120.2	C21—C22—H22C	109.5
C9—C8—C7	120.4 (5)	H22A—C22—H22C	109.5
C9—C8—H8A	119.8	H22B—C22—H22C	109.5
C7—C8—H8A	119.8	C24—C23—C32	119.6 (5)
C8—C9—C10	121.2 (5)	C24—C23—C21	120.2 (4)
C8—C9—H9A	119.4	C32—C23—C21	120.2 (4)
C10—C9—H9A	119.4	C23—C24—C25	121.8 (6)
C9—C10—C5	118.0 (4)	C23—C24—H24A	119.1
C9—C10—C1	123.7 (4)	C25—C24—H24A	119.1
C5—C10—C1	118.3 (4)	C26—C25—C24	120.4 (6)
N1—C11—C1	111.1 (4)	C26—C25—H25A	119.8
N1—C11—C12	108.6 (5)	C24—C25—H25A	119.8
C1—C11—C12	108.9 (4)	C25—C26—C27	120.8 (6)
N1—C11—H11A	109.4	C25—C26—H26A	119.6
C1—C11—H11A	109.4	C27—C26—H26A	119.6
C12—C11—H11A	109.4	C32—C27—C28	117.9 (6)
C11—C12—H12A	109.5	C32—C27—C26	119.6 (5)
C11—C12—H12B	109.5	C28—C27—C26	122.5 (7)
H12A—C12—H12B	109.5	C29—C28—C27	121.6 (6)
C11—C12—H12C	109.5	C29—C28—H28A	119.2
H12A—C12—H12C	109.5	C27—C28—H28A	119.2
H12B—C12—H12C	109.5	C28—C29—C30	119.5 (6)
N1—C13—C14	123.4 (4)	C28—C29—H29A	120.3
N1—C13—H13A	118.3	C30—C29—H29A	120.3
C14—C13—H13A	118.3	C31—C30—C29	121.3 (7)
C19—C14—C15	118.5 (4)	C31—C30—H30A	119.4
C19—C14—C13	119.9 (4)	C29—C30—H30A	119.4
C15—C14—C13	121.5 (4)	C30—C31—C32	121.3 (6)
C14—C15—C16	120.7 (4)	C30—C31—H31A	119.4
C14—C15—H15A	119.7	C32—C31—H31A	119.4
C16—C15—H15A	119.7	C31—C32—C27	118.4 (5)
C15—C16—C17	120.5 (4)	C31—C32—C23	123.8 (5)
C15—C16—H16A	119.8	C27—C32—C23	117.8 (5)
			
C10—C1—C2—C3	0.6 (8)	C16—C17—C18—C19	−2.5 (8)
C11—C1—C2—C3	−176.5 (5)	C20—C17—C18—C19	179.3 (5)
C1—C2—C3—C4	0.6 (9)	C15—C14—C19—C18	1.3 (8)
C2—C3—C4—C5	−0.9 (9)	C13—C14—C19—C18	−178.7 (5)
C3—C4—C5—C6	−179.0 (6)	C17—C18—C19—C14	1.2 (8)
C3—C4—C5—C10	0.0 (8)	C21—N2—C20—C17	178.9 (5)
C4—C5—C6—C7	177.0 (6)	C18—C17—C20—N2	−168.0 (5)
C10—C5—C6—C7	−2.0 (8)	C16—C17—C20—N2	13.9 (9)
C5—C6—C7—C8	0.2 (9)	C20—N2—C21—C22	129.1 (6)
C6—C7—C8—C9	1.8 (8)	C20—N2—C21—C23	−108.7 (5)
C7—C8—C9—C10	−1.9 (8)	N2—C21—C23—C24	−18.5 (7)
C8—C9—C10—C5	0.1 (7)	C22—C21—C23—C24	100.5 (6)
C8—C9—C10—C1	−178.3 (5)	N2—C21—C23—C32	162.1 (5)
C6—C5—C10—C9	1.8 (7)	C22—C21—C23—C32	−78.9 (6)
C4—C5—C10—C9	−177.2 (5)	C32—C23—C24—C25	−1.2 (9)
C6—C5—C10—C1	−179.8 (5)	C21—C23—C24—C25	179.5 (6)
C4—C5—C10—C1	1.2 (7)	C23—C24—C25—C26	0.3 (11)
C2—C1—C10—C9	176.8 (5)	C24—C25—C26—C27	−0.2 (11)
C11—C1—C10—C9	−6.1 (7)	C25—C26—C27—C32	1.0 (10)
C2—C1—C10—C5	−1.5 (7)	C25—C26—C27—C28	−179.5 (7)
C11—C1—C10—C5	175.5 (4)	C32—C27—C28—C29	−0.4 (10)
C13—N1—C11—C1	−147.5 (5)	C26—C27—C28—C29	−180.0 (7)
C13—N1—C11—C12	92.8 (6)	C27—C28—C29—C30	0.1 (11)
C2—C1—C11—N1	−27.7 (7)	C28—C29—C30—C31	−0.1 (10)
C10—C1—C11—N1	155.2 (4)	C29—C30—C31—C32	0.4 (9)
C2—C1—C11—C12	91.8 (6)	C30—C31—C32—C27	−0.7 (8)
C10—C1—C11—C12	−85.2 (6)	C30—C31—C32—C23	−178.5 (5)
C11—N1—C13—C14	−176.5 (6)	C28—C27—C32—C31	0.7 (8)
N1—C13—C14—C19	167.0 (5)	C26—C27—C32—C31	−179.7 (6)
N1—C13—C14—C15	−13.0 (9)	C28—C27—C32—C23	178.7 (5)
C19—C14—C15—C16	−2.4 (9)	C26—C27—C32—C23	−1.8 (8)
C13—C14—C15—C16	177.6 (6)	C24—C23—C32—C31	179.7 (6)
C14—C15—C16—C17	1.1 (9)	C21—C23—C32—C31	−0.9 (8)
C15—C16—C17—C18	1.4 (9)	C24—C23—C32—C27	1.9 (7)
C15—C16—C17—C20	179.5 (6)	C21—C23—C32—C27	−178.8 (5)

### Hydrogen-bond geometry (Å, °)

**Table:**

*D*—H···*A*	*D*—H	H···*A*	*D*···*A*	*D*—H···*A*
C7—H7A···Cg1^i^	0.93	3.29	4.042 (6)	140
C8—H8A···Cg2^i^	0.93	3.14	3.797 (5)	129
C12—H12A···Cg3^ii^	0.96	2.79	3.677 (6)	154
C18—H18A···Cg4^ii^	0.93	2.62	3.520 (5)	163
C20—H20A···Cg5^ii^	0.93	2.98	3.681 (5)	133

Symmetry codes: (i) *x*−1/2, −*y*+3/2, −*z*; (ii) *x*+1/2, −*y*+3/2, −*z*.
